# Development of a Double Antibody Sandwich ELISA for West Nile Virus Detection Using Monoclonal Antibodies against Non-Structural Protein 1

**DOI:** 10.1371/journal.pone.0108623

**Published:** 2014-10-10

**Authors:** Xi-Xia Ding, Xiao-Feng Li, Yong-Qiang Deng, Yong-Hui Guo, Wei Hao, Xiao-Yan Che, Cheng-Feng Qin, Ning Fu

**Affiliations:** 1 Laboratory of Emerging Infectious Diseases and Division of Laboratory Medicine, Zhujiang Hospital, Southern Medical University, Guangzhou, China; 2 Department of Virology, State Key Laboratory of Pathogen and Biosecurity, Beijing Institute of Microbiology and Epidemiology, Beijing, China; Wuhan University, China

## Abstract

The early diagnosis of West Nile virus (WNV) infection is important for successful clinical management and epidemiological control. The non-structural protein 1 (NS1) of flavivirus, a highly conserved and secreted glycoprotein, is abundant in the serum of flavivirus-infected patients and represents a useful early diagnostic marker. We developed a WNV-specific NS1 antigen-capture ELISA using two mouse monoclonal antibodies (MAbs) that recognised distinct epitopes of the NS1 protein of WNV as capture and detection antibodies. The antigen-capture ELISA displayed exclusive specificity to WNV without cross-reaction with other related members of the flavivirus family, including the dengue virus, yellow fever virus, Japanese encephalitis virus, and tick-borne encephalitis virus. Additionally, the specificity was presented as no false positive in normal (0/1003) and DENV-infected (0/107) human serum specimens. The detection limit of the antigen-capture ELISA was as low as 15 pg/ml of recombinant WNV NS1 protein (rWNV-NS1) and 6.1 plaque-forming units (PFU)/0.1 ml of WNV-infected culture supernatant. In mice infected with WNV, the NS1 protein was readily detected in serum as early as one day after WNV infection, prior to the development of clinical signs of the disease. The sensitivity of the NS1 capture ELISA (93.7%) was significantly higher (79.4%) than that of real-time reverse transcription polymerase chain reaction in 63 serum samples from WNV-infected mice (*p* = 0.035). This newly developed NS1 antigen-capture ELISA with high sensitivity and specificity could be used as an efficient method for the early diagnosis of WNV infection in animals or humans.

## Introduction

West Nile virus (WNV), a member of the flavivirus family, is closely related to significant human pathogens such as yellow fever virus (YFV), dengue virus (DENV) type 1 to 4, Japanese encephalitis virus (JEV), tick-borne encephalitis virus (TBEV), St. Louis encephalitis virus (SLEV), and Murray Valley encephalitis virus (MVEV). WNV is transmitted through a mosquito-bird-mosquito route and causes a range of illnesses in humans including mild fever, acute flaccid paralysis, and lethal encephalitis. WNV presents a global threat to human and animal health across the United States and Canada as well as in temperate regions such as Europe and Africa [1]. The public health concern regarding these diseases attracted increasing attention during the last decade in WNV endemic areas of Europe and the Middle East [2]. Since 1999, WNV has spread rapidly throughout North America, causing epidemics of WNV encephalitis, meningitis, and acute flaccid paralysis in naive populations. There were 39,462 cases of WNV infection in the United States from 1999 to 2013, and 1663 deaths have been reported (http://www.cdc.gov/ncidod/dvbid/westnile/index.htm).

A specific therapy or vaccine has not been approved for WNV infection in humans, although passive transfer of specific antibodies and immunoglobulins has been used as a therapeutic attempt [3], [4], [5], and, therefore, an early, specific, and accurate diagnosis of WNV infection might be more important, particularly in regions where multiple flaviviruses co-circulate. The current diagnostic methods for WNV infection include serological tests, viral isolation, virus nucleic acid and infected cell detection. Generally, serological tests and virus nucleic acid detection by RT-PCR are used. RT-PCR requires specialised laboratory equipment and experienced technicians and could result in a false negative because of the short duration of viraemia and low virus titres during human WNV infection [6], [7], [8]. Serological tests, including detection of an antibody and virus protein, are considered specific and convenient, however, there are problems to be resolved. The currently available commercial kits based on IgM antibody detection could be used to identify WNV infection. However, because of the 3∼4 day window phase, the kits might be limited for early diagnosis as a result of the delay between initial infection and seroconversion [9], [10]. Cross-reactivity with other anti-flavivirus antibodies, prior exposure to heterologous flavivirus or vaccinations could interfere with and limit the usefulness of serological diagnostic tests [11]. Therefore, a method for the specific detection of viral antigens for early diagnosis of acute WNV infection is necessary.

The following two commercial immunochromatographic antigen test kits have recently become available for WNV detection in birds: the VecTest WNV antigen assay (Medical Analysis Systems, Inc., Camarillo, CA, USA) [12], [13], [14], [15] and the Rapid Analyte Measurement Platform (RAMP) WNV test (Response Biomedical Corp., Burnaby, BC, Canada) [12],[16]. These commercial tests have been used for rapid WNV antigen detection in mosquito emulsions, oral and cloacal swabs, and feathers obtained from dead crows with high viral copies [14], [16]. The sensitivities of these two assay systems are only 10^5.17^ PFU/ml and 10^3.17^ PFU/ml for viral culture supernatants or 94% and 65% for WNV-positive mosquito pools [12], suggesting that the kits are less useful for screening for WNV infection because of their low sensitivities [14], [16]. Based on the presence of NS1 in the acute-phase sera of DENV and WNV-infected patients and hamsters [17], [18], more attention has been focused on the development of antigen-capture assays for the detection of the NS1 protein of flavivirus. NS1 is a highly conserved glycoprotein that is secreted at high levels and is detected in the serum of flavivirus-infected patients. In dengue-infected patients, the NS1 antigen was found circulating from the first day after the onset of fever up to day 9 after the onset of symptoms [19]. The NS1 antigen-capture assay used in WNV-infected hamsters and mice appears to enable early detection of infection even prior to the appearance of IgM [18], [20]. The detection limit of these two assays are more than 0.5 ng/ml, and the assays appear not to be sensitive enough to enable detection at 3 days after infection, implying that these assays might not be useful for early diagnosis in clinical applications. In addition, the NS1 capture ELISA reported by Macdonald [18] is unable to distinguish between WNV and other flavivirus infections because the antibody used in this assay is directed against cross-reactive antigenic determinants shared by WNV and other flaviviruses. Saxena et al. used a WNV-NS1 polyclonal antibody as the capture antibody, and the inherent batch-to-batch variation ensures that this assay is not amenable to standardisation [21]. To establish the WNV-specific NS1 antigen-capture ELISA assay, we prepared a panel of MAbs and developed a highly sensitive and specific antigen-capture ELISA using two WNV-NS1 MAbs that recognise different epitopes of NS1 as capture and detection antibodies for the early diagnosis of WNV infection.

## Materials and Methods

### Viruses and strains

WNV strain Chin-01 (GenBank accession no. AY490240.2) [22], JEV vaccine strain SA14-14-2 (GenBank accession no. D90195) [23], [24], and TBEV strain An1 were from the Beijing Institute of Microbiology and Epidemiology in China. The four DENV serotype strains (DENV1, Hawaii; DENV2, New Guinea-C; DENV3, Guanxi-80-2; and DENV4, H241) and YFV strain 17D were obtained from the Guangzhou Center for Disease Control and Prevention, Guangzhou, China (Guangzhou CDC) [25], [26], [27]. Slides coated with immobilised WNV-infected cells were used to detect anti-WNV antibodies by an indirect immunofluorescence test (Euroimmun AG, Germany).

### Viral amplification

The cell culture media and supplements were obtained from Invitrogen (Carlsbad, CA, USA). The WNV, JEV, TBEV, and YFV viruses were propagated in BHK21 cells in HEPES-buffered Dulbecco's Modified Eagle Medium (DMEM) supplemented with 50 µg/ml gentamicin and 2% foetal bovine serum and incubated at 37°C in a 5% CO_2_ atmosphere for 3–5 days. The four DENV serotype strains were propagated in C6/36 cells in Eagle's Minimal Essential Media (MEM) supplemented with 50 µg/ml gentamycin and 2% foetal bovine serum and incubated at 33°C for 3–5 days. After the cytopathic effects were observed, the cell culture supernatants were collected and clarified by centrifugation at 1,500 rpm for 10 min. The titre of each virus pool was determined by a plaque assay in Vero-E6 cells, as previously reported [28].

### Serum samples

The WNV-infected mouse models were performed as previously described [22]. Twenty-four-week-old specific-pathogen-free female BALB/c mice were infected intraperitoneally with a 1×10^5^ PFU dose of WNV or JEV or mock infected with phosphate-buffered saline (PBS). The blood samples were collected daily for 24 hr by phlebotomy of the mouse tail veins, centrifuged at 3,000 rpm for 10 min, and frozen at −80°C until use. A total of 107 DENV1-infected patient sera samples were obtained from the Guangzhou CDC [29], [30], [31]. Control sera used for determining the cut-off value differentiating positive from negative samples were obtained from 1003 healthy Chinese volunteers at ZhuJiang Hospital of Southern Medical University, Guangzhou, China.

The experimental procedures involving animals were approved by and conducted in strict accordance with the guidelines of the Animal Experiment Committee of the State Key Laboratory of Pathogen and Biosecurity, Beijing, China. The patient serum samples [25], [29], [30] and control sera was obtained from blood donors, and the patient sera, with the following reference number: ZJYY-2012-JYYXB-002, was obtained from an existing collection approved by the Ethics Committee of the Zhujiang Hospital of Southern Medical University, Guangzhou, China.

### Preparation of recombinant NS1 protein

The expression and purification of the rWNV-NS1 protein from *Escherichia coli* were performed as described previously [32]. The protein concentration was determined using a Bicinchoninic Acid Protein Assay Kit (Sigma-Aldrich, St. Louis, MO, USA), according to the manufacturer's instructions. The rWNV-NS1 protein was identified by Western blot analysis using mouse anti-GST MAb and cross-reactive MAbs of flavivirus [32]. Additionally, the rNS1 proteins of the four DENV serotypes were prepared as described previously in our laboratory [25], [26], [27] and were used for the determination of the specificity and cross-reactivity of WNV-NS1 MAbs.

### Preparation and identification of MAbs against WNV-NS1

The preparation and identification of MAbs against the NS1 protein were performed as described previously, with some modifications [27]. Briefly, 4- to 6-week-old female BALB/c mice were subcutaneously immunised with 50 µg of refolded rWNV-NS1 emulsified with complete Freund's adjuvant (Sigma-Aldrich, USA), followed by three boosts with 30 µg of rWNV-NS1 in incomplete Freund's adjuvant every 10 days by intraperitoneal inoculation. Seven days after the fourth immunisation, the mice were bled, and the serum samples were collected and tested by ELISA with rWVN-NS1 as the coating antigen to determine the antibody titre against rWNV-NS1. The mice that had the highest antibody titre were administered an additional intraperitoneal inoculation with 100 µg of rWNV-NS1 in PBS, and the splenocytes were fused with mouse myeloma cells (NS-1) 3 days later. The hybridoma cells were screened primarily by an indirect ELISA with rWNV-NS1, rGST, or four serotypes of rDENV-NS1 proteins as coating antigens. The supernatant of the screened cells were further confirmed by an indirect immunofluorescence assay (IFA) using slides coated with immobilised WNV-infected cells. Then, the specificity and cross-reactivity of the antibodies were further evaluated by an indirect IFA that detected antibodies binding to C6/36 cells infected with four serotypes of DENV, JEV, and YFV. The positive hybridoma cells were cloned by limited dilution. The MAb isotypes were determined using a commercially available mouse MAb isotyping kit (Zymed Laboratories, Carlsbad, CA, USA). The MAbs were purified from ascitic fluids by the caprylic acid-ammonium sulfate precipitation method and conjugated with horseradish peroxidase (HRP; Sigma-Aldrich, USA) using periodate oxidation.

### Competition ELISA

The binding epitopes of the MAbs were analysed using a competition ELISA with rWNV-NS1 protein as the coating antigen. Microwell plates (Costar Corning, Inc., Corning, NY, USA) were coated with 100 µl/well of rWNV-NS1 at a concentration of 1 µg/ml. After blocking, non-HRP-conjugated MAb at a concentration of 500 µg/ml was incubated with HRP-conjugated MAb at a dilution of 1∶500 at 37°C for 1 hr. After the plates were washed, colour reactions were developed using tetramethylbenzidine (TMB, KPL, Gaithersburg, VA, USA) with hydrogen peroxide (H_2_O_2_) as a substrate solution and stopped with 0.3 N sulphuric acid (100 µl/well); the absorbance was read at 450 nm in an ELISA plate reader (Bio-Tek, Winooski, VT, USA). The influenza virus NS1 protein MAb IVNS1-M6 [33] was used as an irrelevant control. The percentage of inhibition was calculated according to the following formula: [1-(OD_450_ of the test well/OD_450_ of the control well)] ×100%, where OD_450_ is the optical density at a wavelength of 450 nm. If the inhibition were greater than 75%, it was defined as competitive inhibition; if the inhibition were between 75% and 25%, it was defined as relative competition; and if the inhibition were <25%, it was defined as non-competitive inhibition.

### Development of MAb-based WNV-NS1 antigen-capture ELISA

To select the best combination of capture and detection MAbs for a NS1 antigen-capture ELISA, NS1 MAbs were paired according to their affinity to different epitopes of rWNV-NS1. The procedure was performed as described previously [25], with slight modifications. Microplates (Costar, Corning, NY, USA) were coated with 100 µl/well of each capture MAb at a concentration of 10 µg/ml at 4°C overnight. After blocking, a series of diluted inactivated virus-infected culture supernatants and uninfected controls were added to the wells in duplicate and incubated at 37°C for 1 hr. After the plates were washed, the HRP-labelled MAbs were added in a working concentration, and the plates were incubated at 37°C for 30 min. Then, the wells were washed with phosphate-buffered saline Tween-20 (PBS-T), followed by the addition of TMB. The reaction was stopped by the addition of sulphuric acid (0.3 N, 100 µl/well) 10 min later, and the absorbance was measured as described above.

### Detection of NS1 antigen in viral culture supernatants and sera using the NS1 antigen-capture ELISA

To evaluate the sensitivity and specificity of the antigen-capture assay, a series of diluted rWNV-NS1 and rDENV-NS1 proteins starting from a concentration of 500 ng/ml to 1.9 pg/ml were analysed. Bovine serum albumin (BSA) was used to establish the baseline for the assay, and a sample was considered positive if the OD_450_ was two fold greater than that of BSA. The inactivated viral culture supernatants of WNV, JEV, TBEV, YFV, four serotypes of DENV, and an uninfected culture supernatant as the control quantified by a plaque assay were used to evaluate the efficiency of the NS1 antigen-capture ELISA. Because WNV clinical samples are not available in China, samples from WNV-infected mice were subjected to the NS1 antigen-capture ELISA. The groups of BALB/c mice were administered with 1×10^5^ PFU of WNV, and sera were collected on days 1, 2, 3, 4, 5, 6, and 7 post infection. Mice infected with JEV or mock infected with PBS were set as the controls. All the infected animal sera were serially diluted 100-fold in PBS, and the normal human sera were serially diluted 10-fold in PBS. The procedures for the NS1 antigen-capture ELISA were as described above. The cut-off value was set as the average value of the control sera plus five times the standard deviation (SD), and a sample was considered positive if it yielded an OD_450_ value above the cut-off value. The end point dilutions of the detectable NS1 in the viral culture supernatants were defined as the highest dilution that showed an OD_450_ value two fold greater than that of the uninfected culture supernatants.

### Real-time RT-PCR assay

For the quantitation of viral RNA in the WNV-infected mouse serum samples, the viral RNA was harvested from 20 µl of serum using a QIAamp Viral RNA Mini Kit (Qiagen, Valencia, CA, USA). The average number of WNV RNA copies in each sample was determined from 5 µl of extract in duplicate by a real-time RT-PCR assay using a Taqman fast virus one-step Master Mix kit (Life Technologies, Foster City, CA, USA) and fluorescent primer probe sets for the WNV E gene [34].

### Statistical Analysis

The data were analysed with Prism software (Graph-Pad Software). The statistical data were calculated using SPSS statistical package version 20. The chi-squared test was used to evaluate the statistical significance of the results between the WNV-NS1 ELISA and the real-time RT-PCR assay. A *P* value of <0.05 was considered statistically significant for all the parameters.

## Results

### Selection and characterisation of capture and detection MAbs for WNV-NS1 antigen-capture ELISA

**Figure 1:**
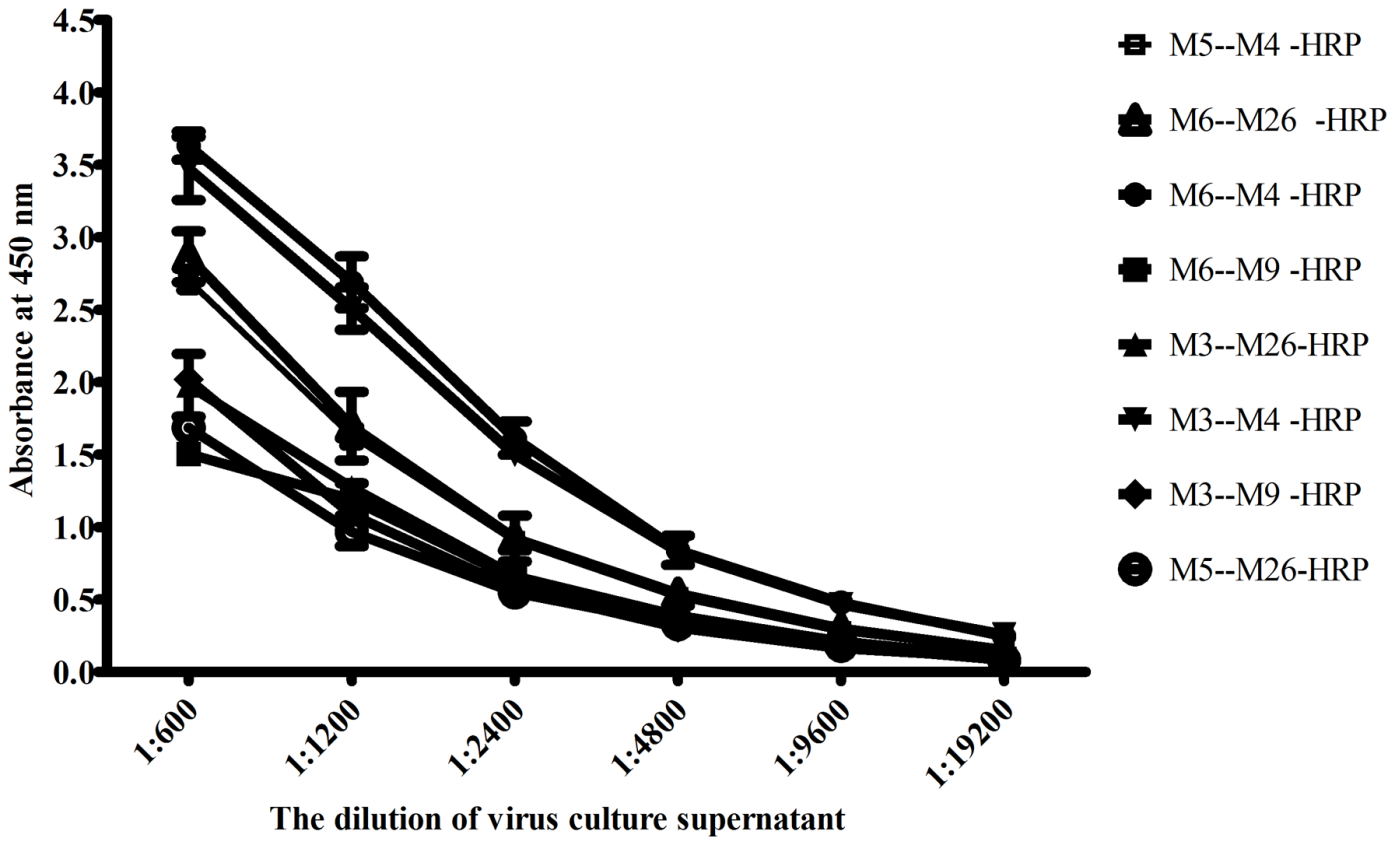
The sensitivity of the double antibody sandwich ELISA using different capture and detection MAbs pairs. The detection of NS1 in culture supernatants of WNV-infected cells by different MAb pairs. The culture supernatants of WNV-infected cells were serially diluted 2-fold. Data points represent the mean ± standard deviation from three replicates. The most effective pairs were determined by the highest dilution of the OD_450_ values.

A total of 65 hybridoma cell clones that produced MAbs against WNV-NS1 protein were established based on their strong positive reactivity with the rWNV-NS1 protein in the ELISA and the WNV-infected cells in the IFA. The specificity of the MAbs was further identified by excluding their cross-reactivity to the four serotypes of DENV, JEV, and YFV using IFA and the four serotypes of DENV-NS1 protein using ELISA. The IgG isotype determination revealed that the 65 MAbs consisted of IgG1 (*n* = 46), IgG2a (*n* = 8), IgG2b (*n* = 9), and IgG3 (*n* = 2). The characteristics of these MAbs are shown in **Table 1**. Of the 65 MAbs, named WNVNS1-M1 to WNVNS1-M65, 39 MAbs specifically reacted with WNV-NS1 without cross-reactivity with the four serotypes of DENV, JEV, and YFV in either ELISA or IFA, indicating that the 39 MAbs specific for WNV could be useful for developing a WNV-NS1-specific antigen-capture ELISA. The remaining 26 MAbs exhibited cross-reactivity with the four serotypes of DENV and JEV in different cross-reactive patterns. The sensitivity of the sandwich formation requires a pair of antibodies that are capable of binding to discrete, non-overlapping epitopes of the antigen [35]. Thus, the epitopes recognised by these MAbs were determined by a competition ELISA with rWNV-NS1 as the coating antigen. The 39 anti-rWNV-NS1 MAbs were divided into at least 13 groups, and each group reacted with the identical epitope or sterically overlapped the rWNV-NS1 epitopes. All of the 39 MAbs specific to WNV-NS1 were paired according to their abilities to recognise distinct epitopes of NS1. The eight most effective pairs of capture and detection MAbs that recognised different epitopes were primarily selected based on their sensitivity to detect the WNV-NS1 protein (**Fig. 1**). The MAb pair of immobilised M6 and HRP-M4 was demonstrated to be the most effective to detect WNV among the eight pairs of MAbs. A checkerboard analysis of the serial dilutions of the capture and detection antibodies was conducted to optimise the reaction conditions of the antigen-capture assay. The optimal concentration for the capture by MAb M6 was determined as 10 µg/ml and for detection by MAb HPR-M4 was 1∶1000.

**Table 1:** Characteristics of MAbs against the NS1 protein of WNV.

Hybridoma (original clone no.)	Isotype	Epitope group	ELISA result^a^:					IFA result^b^:						
			WNV	DENV1	DENV2	DENV3	DENV4	WNV	DENV1	DENV2	DENV3	DENV4	JEV	YFV
WNVNS1-M1	IgG1	I	+++	-	-	-	-	++	-	-	-	-	-	-
WNVNS1-M2	IgG1	XIII	+++	-	-	-	-	+	-	-	-	-	-	-
WNVNS1-M3	IgG1	II	+++	-	-	-	-	+	-	-	-	-	-	-
WNVNS1-M4	IgG1	III	+++	-	-	-	-	++	-	-	-	-	-	-
WNVNS1-M5	IgG1	II	+++	-	-	-	-	+	-	-	-	-	-	-
WNVNS1-M6	IgG1	II	+++	-	-	-	-	+	-	-	-	-	-	-
WNVNS1-M7	IgG3	IV	+++	-	-	-	-	+	-	-	-	-	-	-
WNVNS1-M8	IgG1	VIII	+++	-	-	-	-	+	-	-	-	-	-	-
WNVNS1-M9	IgG1	III	++	-	-	-	-	++	-	-	-	-	-	-
WNVNS1-M10	IgG1	IV	++++	-	-	-	-	+	-	-	-	-	-	-
WNVNS1-M11	IgG1	V	++++	-	-	-	-	++	-	-	-	-	-	-
WNVNS1-M12	IgG2a	VII	++++	-	-	-	-	++	-	-	-	-	-	-
WNVNS1-M13	IgG1	X	+++	-	-	-	-	+	-	-	-	-	-	-
WNVNS1-M14	IgG3	VI	+++	-	-	-	-	+	-	-	-	-	-	-
WNVNS1-M15	IgG2b	IV	+++	-	-	-	-	+	-	-	-	-	-	-
WNVNS1-M16	IgG2b	VII	+++	-	-	-	-	+	-	-	-	-	-	-
WNVNS1-M17	IgG1	IX	+++	-	-	-	-	+	-	-	-	-	-	-
WNVNS1-M18	IgG1	IV	++++	-	-	-	-	++	-	-	-	-	-	-
WNVNS1-M19	IgG1	XII	+++	-	-	-	-	+	-	-	-	-	-	-
WNVNS1-M20	IgG1	IV	+++	-	-	-	-	++	-	-	-	-	-	-
WNVNS1-M21	IgG1	IV	+++	-	-	-	-	+	-	-	-	-	-	-
WNVNS1-M22	IgG1	IV	+++	-	-	-	-	++	-	-	-	-	-	-
WNVNS1-M23	IgG1	IV	++++	-	-	-	-	+	-	-	-	-	-	-
WNVNS1-M24	IgG1	XI	+++	-	-	-	-	+	-	-	-	-	-	-
WNVNS1-M25	IgG1	VI	+++	-	-	-	-	+	-	-	-	-	-	-
WNVNS1-M26	IgG1	III	++	-	-	-	-	+++	-	-	-	-	-	-
WNVNS1-M27	IgG1	XII	++++	-	-	-	-	+	-	-	-	-	-	-
WNVNS1-M28	IgG1	VII	+++	-	-	-	-	+	-	-	-	-	-	-
WNVNS1-M29	IgG2b	VII	++++	-	-	-	-	++	-	-	-	-	-	-
WNVNS1-M30	IgG1	VI	+++	-	-	-	-	++	-	-	-	-	-	-
WNVNS1-M31	IgG2a	VII	+++	-	-	-	-	+	-	-	-	-	-	-
WNVNS1-M32	IgG1	VI	++++	-	-	-	-	++	-	-	-	-	-	-
WNVNS1-M33	IgG2b	VII	++++	-	-	-	-	+	-	-	-	-	-	-
WNVNS1-M34	IgG	VII	+++	-	-	-	-	+∼+	-	-	-	-	-	-
WNVNS1-M35	IgG2b	VII	++++	-	-	-	-	++	-	-	-	-	-	-
WNVNS1-M36	IgG1	VI	+++	-	-	-	-	+	-	-	-	-	-	-
WNVNS1-M37	IgG1	VI	++++	-	-	-	-	+	-	-	-	-	-	-
WNVNS1-M38	IgG1	VII	+++	-	-	-	-	++	-	-	-	-	-	-
WNVNS1-M39	IgG1	V	+++	-	-	-	-	++	-	-	-	-	-	-
WNVNS1-M40	IgG1	ND^c^	+++	-	++++	-	+++	++	-	-	-	-	-	-
WNVNS1-M41	IgG2a	ND	++++	-	-	-	-	+	-	-	-	-	-	-
WNVNS1-M42	IgG2b	ND	+++	-	-	-	-	+	-	-	-	-	-	+
WNVNS1-M43	IgG1	ND	+++	-	-	-	-	+	-	-	-	-	-	+
WNVNS1-M44	IgG1	ND	+++	-	-	-	-	+	-	-	-	-	-	+
WNVNS1-M45	IgG1	ND	+++	-	-	-	-	+	-	+	+	+	-	-
WNVNS1-M46	IgG2a	ND	+++	-	++++	-	+++	+	-	+++	-	-	-	++
WNVNS1-M47	IgG2a	ND	+++	-	++++	-	+++	++	-	+++	-	-	-	+++
WNVNS1-M48	IgG2b	ND	+++	-	+++	-	+++	++	-	++	-	-	-	+++
WNVNS1-M49	IgG1	ND	+++	-	-	-	-	+++	+	+	+	+	-	-
WNVNS1-M50	IgG1	ND	+++	+++	+++	++++	++++	+	+	+	-	+	-	+++
WNVNS1-M51	IgG1	ND	+++	-	+++	-	-	+	-	+	-	-	-	++
WNVNS1-M52	IgG2a	ND	+++	-	++++	-	++++	++	-	++	-	-	-	++
WNVNS1-M53	IgG2b	ND	+++	-	+++	-	+++	++	-	+++	-	-	-	++
WNVNS1-M54	IgG2a	ND	++++	-	++++	-	+++	++	-	++	-	-	-	++
WNVNS1-M55	IgG1	ND	+++	-	-	-	-	+	-	-	-	-	-	+
WNVNS1-M56	IgG2a	ND	+++	-	++	-	+++	+	-	+	-	+	-	+
WNVNS1-M57	IgG1	ND	+++	-	-	-	-	++	-	-	-	-	-	++
WNVNS1-M58	IgG1	ND	+++	-	+++	-	+++	+	-	+++	-	-	-	+++
WNVNS1-M59	IgG1	ND	++++	-	-	-	-	++	-	-	-	+++	-	++
WNVNS1-M60	IgG1	ND	++++	-	-	-	-	++	-	-	-	-	-	+
WNVNS1-M61	IgG2b	ND	+++	-	-	-	-	+	-	-	-	-	-	++
WNVNS1-M62	IgG1	ND	+++	-	-	-	-	++	-	-	-	-	-	+
WNVNS1-M63	IgG1	ND	+++	-	-	-	-	+	-	-	-	+++	-	++
WNVNS1-M64	IgG1	ND	++++	-	-	-	-	+	-	-	-	-	-	+
WNVNS1-M65	IgG1	ND	+++	-	-	-	-	+	-	-	-	-	-	+

a Purified recombinant WNV-NS1 protein and DENV-NS1 were used as the coating antigen and were reacted with purified MAbs against the NS1 protein. The absorbance was measured at 450 nm: +, OD_450_ = 0.5 to 1; ++, OD_450_ = 1 to 2; +++, OD_450_ = 2 to 3; OD_450_>3.

b The reactivity of each MAb was determined by IFA with WNV, DENVs, YFV, and JEV, and their IFA reactivity with normal cells were negative (data not shown). −, no reactivity; +, weak reactivity; ++, intermediate reactivity; +++, strong reactivity.

c Not detected.

### Specificity and sensitivity of the WNV-NS1 antigen-capture ELISA

**Figure 2:**
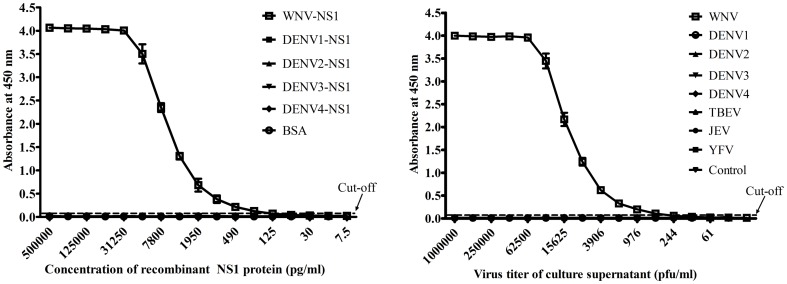
Evaluation of the sensitivity and specificity of the rWNV-NS1 antigen-capture ELISA. A, The NS1 standard curve as determined by the purified rWNV-NS1 antigen-capture ELISA. Various concentrations of WNV-NS1 and DENV1-4-NS1 proteins were analysed. BSA was used to establish the baseline. B, The detection of NS1 in WNV-infected cell culture supernatants and other closely related members of the flavivirus family, DENV, JEV, YFV, and TBEV, by the NS1 antigen-capture ELISA. Two-fold serially diluted supernatants from WNV-, four DENV serotype-, JEV-, YFV-, or TBEV-infected cells were subjected to the WNV-NS1 antigen-capture ELISA. Data points represent the mean ± standard deviation from three replicates. The cut-off value was set at twice the average value of the negative controls from three replicates. Positive OD450 values were only observed for the WNV-NS1 protein and WNV-infected cell supernatants.

To evaluate the detection limit and specificity of the antigen-capture ELISA, the 2-fold serially diluted rWNV-NS1 and rDENV-NS1 proteins were tested in duplicate. BSA was used to establish the baseline. The detection limit of the rWNV-NS1 protein was approximately 15–30 pg/ml, and the linear portion of the standard curve ranged from 15.62 to 125 ng/ml (**Fig. 2A**). There was no positive result with the rDENV-NS1 protein. The specificity and sensitivity of the WNV-NS1 capture ELISA was further assessed by testing for the presence of NS1 in the culture supernatants of WNV, four serotypes of DENV, JEV, YFV, and TBEV (**Fig. 2B**). Only the WNV-infected cell culture supernatants presented a positive signal. The sensitivity of the assay was approximately 6.1 PFU/0.1 ml for the WNV-infected supernatants, and no positive results or cross-reactivity were observed for the four serotypes of DENV, JEV, YFV, or TBEV. These results indicate that the NS1 antigen-capture assay is specific for the detection of WNV.

### Validation with clinical samples

**Figure 3:**
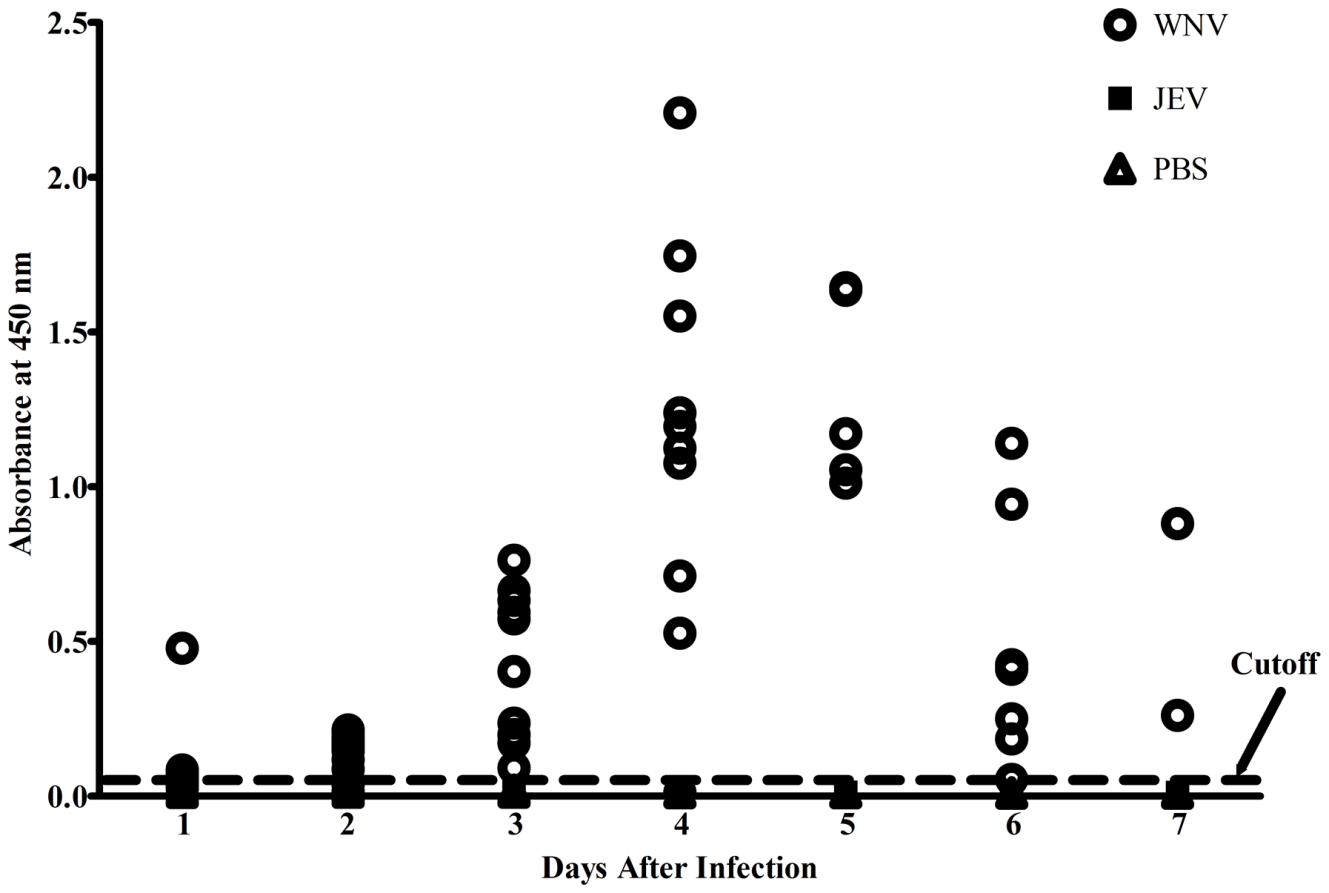
The detection of NS1 in WNV-infected mice sera by the NS1 antigen-capture ELISA. The data represent the OD_450_ of the serum samples at 1∶100 dilution. The dashed line represents the cut-off value, which is twice the mean value of the negative control. Each data point represents the mean OD_450_ of duplicate assays. The results were considered positive if a sample yielded an OD_450_ above the cut-off value. The cut-off value was set as twice the mean value of the negative control. Each data point represents the mean OD_450_ of duplicate assays.

Using this assay, the soluble NS1 was reliably detected starting from day 1 after the WNV infection in mice, with peak NS1 concentration at day 4, whereas the NS1 protein began to decrease on day 6 (**Fig. 3**). On days 1 and 3 after infection, NS1 was detected in 85.7% (12/14) and 100% (11/11) of the serum samples among the WNV-infected mice and was not detected in the JEV-infected and mock PBS-infected mice. Additionally, the sensitivity of the WNV-NS1 capture ELISA was compared to the real-time RT-PCR assay in 63 serum samples from 14 WNV-infected mice (**See** **Table 2**). The WNV-NS1 capture ELISA displayed a markedly greater sensitivity with the WNV-infected mice sera, and variations in sensitivity between the antigen-capture ELISA and the real-time RT-PCR were statistically significant as revealed by chi-squared tests (*P* = 0.035).

**Table 2:** Comparison of the sensitivities of the WNV-NS1 ELISA and real-time RT-PCR for the detection of WNV-infected mice serum samples.

Day after WNV infection	No. of serum samples	No. (%) of samples positive by:	
		WNV-NS1 ELISA	Real-time RT-PCR
1	14	12 (85.7)	14 (100)
2	14	13 (92.9)	14 (100)
3	11	11 (100)	11 (100)
4	10	9 (90)	8 (80)
5	5	5 (100)	3 (60)
6	7	7 (100)	0 (0)
7	2	2 (100)	0 (0)
Total	63	59 (93.7)	50 (79.4)

To establish the baseline of the WNV-NS1 antigen-capture assay for the clinical evaluation, the control sera from 1003 healthy Chinese volunteers were analysed. The mean OD_450_ value of these sera was 0.01, with a SD of 0.0031. Next, the cut-off value was set as follows: 0.01+0.0031×5 = 0.0253. By these criteria, none of the 1003 sera samples from healthy blood donors was defined as positive. Thus, the detection of the WNV-NS1 protein had a specificity of 100% (1003 of 1003).

A total of 107 serum specimens collected from patients during the DENV-1 epidemic in Guangdong in 2006, which were confirmed as DENV1-NS1 antigen-positive by our previous study [25], were examined using this assay. All of the specimens were negative in this assay, indicating the specificity of the WNV-NS1 antigen-capture ELISA.

## Discussion

Because of the cross-reactivity of anti-flavivirus antibodies, prior exposure to heterologous flavivirus or vaccinations could limit the utility of serological diagnostic tests [11]. However, there has been no effective commercial antigen detection kit for WNV infection in humans. Macdonald and Saxena have developed two NS1 antigen-capture ELISAs, however, these assays do not effectively distinguish WNV from other flavivirus infections or standardization because the antibodies used in these assays are either a flavivirus NS1 protein cross-reactive MAb or a WNV-NS1 polyclonal antibody. These results in poor specificity in detecting WNV or intra- and inter- laboratory variability caused by batch-to-batch variations in polyclonal antisera. Compared to a polyclonal antibody, the advantages of a MAb-based assay are stability and specificity for diagnosis and research because MAbs from stable hybridoma clones could ensure a continuous supply of large quantities of well characterised antibodies against unique epitopes. The assay system in our work is based on two MAbs recognising different epitopes of the WNV-NS1 protein.

In this study, 65 MAbs against WNV-NS1 were produced by immunising mice with rWNV-NS1. The reaction profile of each antibody was characterised by indirect ELISA and IFA. A total of 26 of 65 MAbs showed cross-reactivity with the four serotypes of DENV and JEV in different cross-reactive patterns, whereas the other 39 MAbs were found to be specific to WNV-NS1 only. These MAbs specifically recognised at least 13 different epitopes of the NS1 protein of WNV, allowing the selection of an optimal pair of MAbs for constructing a double antibody sandwich ELISA. The matched pair of MAb M6 as the capture antibody and MAb M4 as the detection antibody exhibited specific binding to the WNV-infected culture supernatant with a detection limit as low as 6.1 PFU/0.1 ml, without cross-reactivity with the other closely related members of the flavivirus family such as DENV, JEV, YFV, and TBEV. The detection limit of the antigen-capture ELISA was as low as 15 pg/ml, which was much more sensitive than that of 5 ng/ml in the WNV-NS1 ELISA reported by Saxena [21] and 0.5 ng/ml reported by Chung KM [20]. By our assay system, WNV-NS1 sera could be detected at day 1 after infection, compared with day 3 [20]. This high sensitivity and specificity of our ELISA system might be attributed to the sandwich construction paired with the two MAbs selected from 39. We hypothesise that a polyclonal capture antibody might not have high enough affinity to target predominant epitopes of NS1 because of steric effects, thus decreasing the sensitivity of the assay. In addition, an antigen assay based on polyclonal antibodies is subject to intra- and inter-laboratory variability caused by batch-to-batch variations resulting from the use of antisera from different animals, which might be unfavourable to the stability of a detection system and development of a commercial kit. MAbs from stable hybridoma clones ensure a continuous supply of large quantities of well- characterised antibodies that could be easily standardised among different laboratories, which could be developed into a commercial kit in the future. With its high sensitivity in detecting WNV-NS1, the NS1 antigen-capture ELISA might diagnose WNV infection caused by a low viraemia level. This NS1 antigen-capture ELISA was able to detect the WNV antigen of sera in infected mice as early as one day after WNV infection. The detectable NS1 antigen concentration peaked at day 4 and then declined from day 6. The decrease of detectable NS1 in mice sera is likely caused by the production of a specific anti-NS1 antibody, which forms an antibody-antigen complex, thus diminishing the levels of free NS1 and reducing the consequent sensitivity of the antigen-capture ELISA. This finding is in accordance with a previous study in which dissociated NS1 immune complexes extend the window of detection [20]. The positive detection rates of WNV-NS1 increased from 85.7% at 1–4 days to 100% at 5–7 days post infection among the WNV-infected mice serum samples, whereas the serum samples from the JEV-infected and PBS-treated mice were negative. Additionally, the NS1 antigen-capture ELISA displayed greater sensitivity than that of real-time RT-PCR from 1 to 7 days in WNV-infected mouse serum samples (*P* = 0.035), which might be attributed to the short duration of viraemia and low viral RNA titres after WNV infection [6], [7], [8]. For this reason, RT-PCR could not successfully detect WNV-infected sera during the whole process of murine infection, whereas the antigen-capture ELISA could do so.

Our antigen-capture ELISA did not cross-react with the supernatants of four serotypes of DENV, JEV, YFV, or TBEV-infected cell cultures and the sera of DENV1-infected patient. This WNV-NS1 antigen-capture ELISA shows a sensitivity of 6.1 PFU/0.1 ml in culture supernatants and 15 pg/ml for the WNV-NS1 protein as well as a specificity of 100% for the virus culture supernatants and serum samples, which resulted in early detection on day 1 after the infection of mice. Because there is no WNV-infected human serum specimen in China, we made the mock WNV-infected human serum specimens by adding WNV that had been quantified by plaque assay into normal human serum specimens and 37°C incubation for 1 hr, with 2-fold serial dilutions of the serum samples for the analysis. This antigen-capture ELISA measured the mock WNV-infected human serum with a limit of detection of 48.8 PFU/0.1 ml, which was approximately 8 fold lower than that of the WNV-infected culture supernatant. This finding might be attributed to the differential substrate environments of the serum and culture supernatants or some of the NS1 epitopes might have been blocked by the immunoglobulins of the human serum. It could be possible that our NS1-capture ELISA system with high specificity and sensitivity would provide an efficient tool for the detection of WNV infections with early diagnosis, particularly in some regions, such as Asia and North America where there is a high incidence of WNV infection and the population is vaccinated against other flaviviruses (JEV, YFV, and TBEV), rendering serological tests difficult to interpret [36]. Hu et al. proposed that an accurate diagnosis of an acute DENV infection requires a combination of several tests performed at different stages of the disease [31]. It is necessary to combine our NS1-capture ELISA and the IgM antibody test to enhance diagnostic effectiveness, which would be expected to be further evaluated with WNV-infected clinical serum samples in the future.
